# Everolimus and/or Nivolumab-Associated Cytomegalovirus Colitis in a Patient with Metastatic Renal Cell Carcinoma

**DOI:** 10.5152/eurasianjmed.2022.20300

**Published:** 2022-02-01

**Authors:** Rashad Ismayilov, Oktay Halit Aktepe, Konul Sardarova, Can Berk Leblebici, Mustafa Erman

**Affiliations:** 1 Department of Internal Medicine, Hacettepe University Faculty of Medicine, Ankara, Turkey; 2 Department of Medical Oncology, Hacettepe University Cancer Institute, Ankara, Turkey; 3 Department of Pathology, Hacettepe University Faculty of Medicine, Ankara, Turkey; 4 Department of Preventive Oncology, Hacettepe University Cancer Institute, Ankara, Turkey

**Keywords:** Colitis, cytomegalovirus, everolimus, nivolumab, renal cell carcinoma

## Abstract

Everolimus-associated cytomegalovirus colitis is very rare in cancer patients. We present a case of cytomegalovirus colitis that occurred on using everolimus in a 64-year-old male with metastatic renal cell carcinoma who received pazopanib, nivolumab, and everolimus treatments, respectively. Although an increasing number of nivolumab-related cytomegalovirus colitis cases are reported recently, its mechanism of development is still unknown. Our study highlights that clinicians should remember cytomegalovirus reactivation in the presence of diarrhea or colitis in patients receiving everolimus and/or nivolumab. Further studies are needed to elucidate the relationship between immune checkpoint inhibitors and cytomegalovirus reactivation, and these will also be a guide to prevent other possible viral infections.

## Introduction

Everolimus is an orally administered inhibitor of the mammalian target of rapamycin (mTOR) and used in breast cancer, some neuroendocrine tumors, and renal cell carcinoma (RCC) as an antineoplastic agent. Common side effects of the drug are stomatitis, rash, fatigue, anemia, hyperlipidemia, and hyperglycemia.^1^ Everolimus is also used in the prophylaxis of organ rejection in renal and liver transplant patients. Many clinical studies have shown that everolimus reduces the risk of cytomegalovirus (CMV) infection in transplant recipients because CMV replication is dependent on mTOR activity.^2^ However, CMV-related infections are encountered occasionally in patients taking everolimus. We present a case of severe CMV colitis after the use of everolimus in a patient with metastatic RCC and discuss the possible causes.

## Case Presentation

Sixty-four-year-old male with RCC (chromophobe subtype with sarcomatoid differentiation, pT3aN1), who underwent right nephrectomy and received 6 months of pazopanib, was commenced nivolumab treatment (260 mg every 2 weeks) for progressive lung metastases. Significant progression was observed in lung lesions after taking 6 doses of nivolumab. Then the treatment switched to everolimus 10 mg/day, but after 24 days, he had severe abdominal pain, diarrhea 16 times a day, and hematochezia. He had no such gastrointestinal symptoms during the treatment with pazopanib and nivolumab. On physical examination, abdominal tenderness was positive without rebound tenderness and guarding. Laboratory investigation revealed white blood cell (WBC) count of 6.5 × 10^3^ µL (4.3-10.3 × 10^3^ µL) with 21.8% monocyte (5.1-10.9%), hemoglobin 11.7 g/dL (13.6-17.2 g/dL), erythrocyte sedimentation rate (ESR) 62 mm/h, C-reactive protein (CRP) 18.8 mg/dL (0-0.8 mg/dL), and procalcitonin 0.38 ng/mL (0-0.1 ng/mL). Serum sodium and creatinine were 126 mEq/L (136-146 mEq/L) and 1.28 mg/dL (0.67-1.17 mg/dL), respectively. Complete urine analysis was normal and there was no growth in blood and urine cultures.

Abundant leukocytes and erythrocytes were observed in stool microscopy. In the stool culture and polymerase chain reaction (PCR) examination, *Norovirus*, *Clostridium difficile*, *Salmonella*, *Shigella*, *Yersinia*, and *Campylobacter* were not found. No pathological finding was detected in upper endoscopy. In colonoscopy, the entire colon mucosa from the rectum to the cecum was highly hyperemic, congestive, and locally erythematous. The vascular structure was lost and mucous exudated areas were observed in the mucosa, and multiple biopsies were taken from the colon.

**Figure 1.**
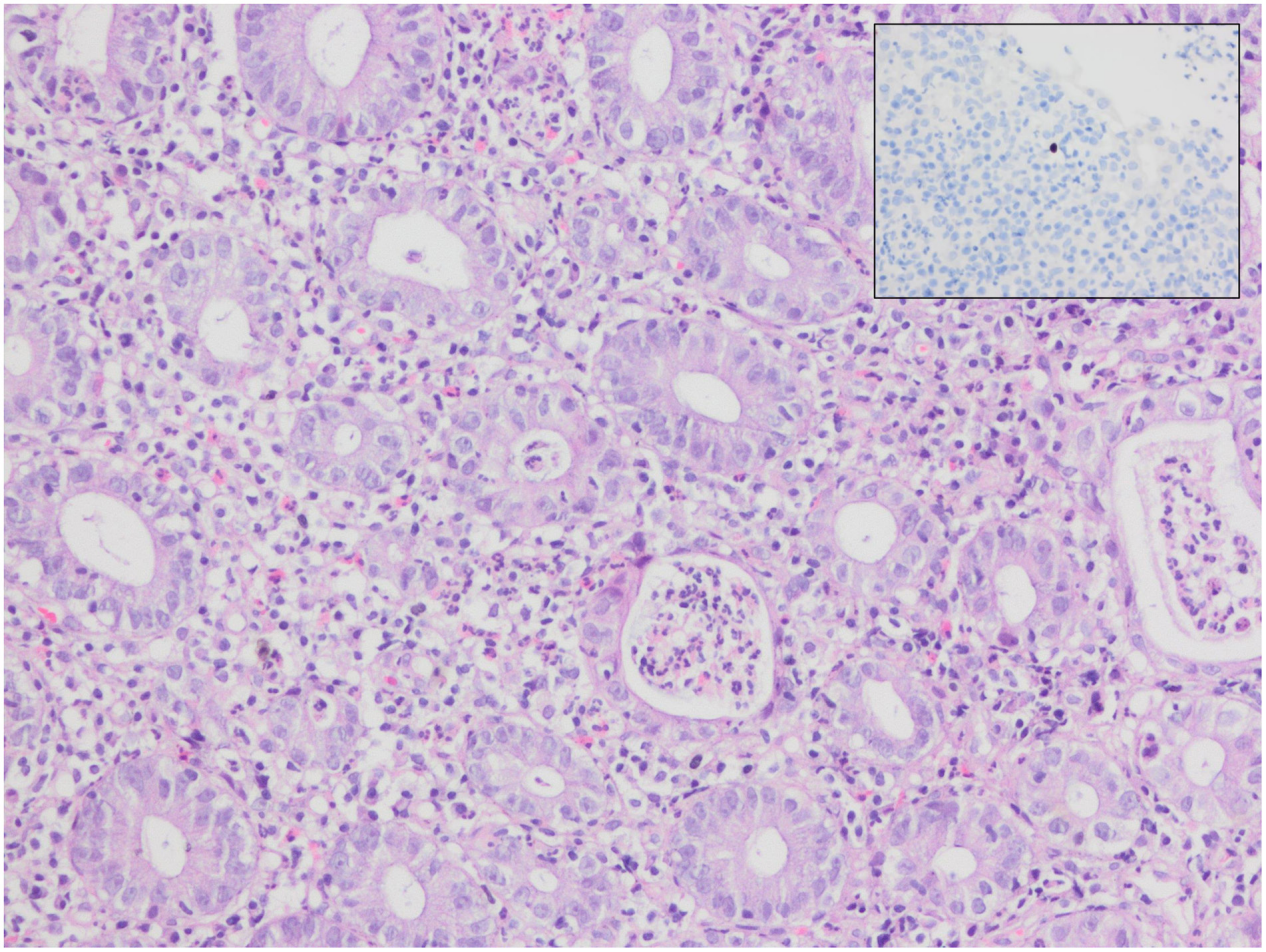
In sections, colon mucosa with ulcerated surface is observed. Active chronic inflammation rich in neutrophils and cryptic abscesses was detected in the lamina propria. Inset shows a small number of immunohistochemical CMV inclusions.

The patient was evaluated to have drug-related colitis and everolimus was discontinued. Methylprednisolone was commenced at a dose of 1 mg/kg per day. On the seventh day of treatment, diarrhea decreased to 12 times per day. In the colonoscopic biopsy, diffuse active colitis and CMV inclusions were observed and CMV was positive as a result of the immunohistochemical study (Figure 1). Cytomegalovirus DNA was detected to be 192 copies in the blood analysis. Subsequently, ganciclovir was initiated intravenously with a dose of 5 mg/kg twice daily and methylprednisolone was tapered and stopped. The patient’s abdominal pain improved gradually and diarrhea decreased to 3 times a day on day 15 of the treatment. On the 15th day of ganciclovir treatment, ESR was 53 mm/h, CRP was 3.36 mg/dL, and procalcitonin was 0.07 ng/mL. Afterward, antiviral therapy was switched to oral valganciclovir 900 mg^BID^ and continued for 3 weeks.

Written informed consent was obtained from the patient.

## Discussion

Renal cell carcinoma accounts for about 80% of all kidney cancers, and the most common subtype is clear cell RCC (ccRCC). Approximately 80% of non-ccRCCs were papillary and chromophobe subtypes. Everolimus is involved in the first-line systemic treatment of metastatic chromophobe non-ccRCCs.^3^ It causes grade 3-4 diarrhea (greater than 7 times stools per day and may require hospitalization) in only 1% of metastatic RCC patients, and everolimus-associated colitis is extremely rare.^1^ The relationship between mTOR inhibitors and CMV infections was studied extensively. It was shown that everolimus suppresses the DNA synthesis of the virus, prevents the spread of CMV infection, and CMV-specific CD8^+^/CD4^+^ T cell response increases in transplant patients treated with everolimus.^2,4^ However, a case of everolimus-related CMV colitis has been previously reported in a patient with metastatic breast cancer.^5^ In the reported case, severe CMV colitis occurred in the third month of everolimus treatment and relapsed when everolimus was restarted after antiviral therapy. Differently, in our patient, gastrointestinal symptoms began on the 24th day.

Nivolumab is a programmed death 1 inhibitor and immune colitis due to such immune checkpoint inhibitors is a well-defined side effect.^6^ Grade 3-4 diarrhea and colitis due to nivolumab are encountered at rates of 0.3% and 0.7%, respectively.^7^ The interval between the initiation of immune checkpoint inhibitors and the onset of diarrhea ranges from 1 to 32 weeks with a median of 8 weeks.^8^ A growing number of cases of steroid and anti-TNF refractory diarrhea associated with immune checkpoint inhibitors have been reported, which were subsequently discovered to be CMV colitis.^9-12^ Recently, Medicines and Healthcare products Regulatory Agency has announced that CMV screening should be performed if patients on nivolumab present with diarrhea or colitis and for patients with steroid-refractory immune-related colitis; use of an additional immunosuppressive agent should only be considered if CMV PCR is negative on biopsy.^13^ The exact mechanism of CMV reactivation in immune checkpoint inhibitors use is still unknown. Immunosuppressive treatments used for immune-related colitis may also lead to infection. In the presented case, CMV reactivation may be secondary to immune activation caused by checkpoint blockade or to immunosuppression caused by progressive metastatic cancer.

Consequently, anticancer drugs can lead to life-threatening situations. Therefore, knowing the side effects well will prevent treatment delays and undesirable results. Cytomegalovirus reactivation should be considered in the presence of diarrhea or colitis in patients using everolimus and/or nivolumab.
